# Implementing of infrared camouflage with thermal management based on inverse design and hierarchical metamaterial

**DOI:** 10.1515/nanoph-2023-0067

**Published:** 2023-04-13

**Authors:** Xinpeng Jiang, Huan Yuan, Xin He, Te Du, Hansi Ma, Xin Li, Mingyu Luo, Zhaojian Zhang, Huan Chen, Yang Yu, Gangyi Zhu, Peiguang Yan, Jiagui Wu, Zhenfu Zhang, Junbo Yang

**Affiliations:** Center of Material Science, College of Sciences, National University of Defense Technology, Changsha 410073, China; Peter Grünberg Research Centre, College of Telecommunications and Information Engineering, Nanjing University of Posts and Telecommunications, Nanjing 210003, China; College of Physics and Optoelectronic Engineering, Shenzhen University, Shenzhen 518060, China; College of Electronic and Information Engineering, Southwest University, Chongqing 400715, China

**Keywords:** hierarchical metamaterial, infrared camouflage, inverse design, multilayer, selective emitter, thermal management

## Abstract

Infrared camouflage is an effective technique to avoid many kinds of target detection by detectors in the infrared band. For a high-temperature environment, thermal management of selective emission is crucial to dissipate heat in the mid-infrared non-atmospheric window (5–8 μm). However, it still remains challenges for balancing infrared camouflage and thermal management. Here, we experimentally demonstrate a multilayer film structure (MFS) for infrared camouflage with thermal management. Combining the ideal emission spectrum and genetic algorithm (GA), the inverse-design MFS containing 7 layers of five materials (SiO_2_, Ge, ZnS, Pt and Au) has been designed. Based on the hierarchical metamaterial, the optimized MFS has high performance of infrared camouflage to against the lidar detection in the near-infrared band. The experimental results reveal the high compatible efficiency among thermal camouflage (*ε*_3–5μm_ = 0.21, *ε*_8–14μm_ = 0.16), laser stealth (*ε*_1.06μm_ = 0.64, *ε*_1.55μm_ = 0.90, *ε*_10.6μm_ = 0.76) and thermal management (*ε*_5–8μm_ = 0.54). Therefore, the proposed MFSs are attractive as basic building block of selective emitter, for the application of advanced photonics such as radiative cooling, infrared camouflage, and thermal emission.

## 1 Introduction

In recent years, promoted by the progress of infrared (IR) detection, IR camouflage has attracted more and more attention [1–4]. Since most military weapons are equipped with IR detection it allow people to observe at night like an owl, there is an extremely urgent need for thermal camouflage to conceal the targets in the IR camera in the military field [5, 6]. According to the Stefan–Boltzmann law (*j* = *εσT*^4^), the intensity of the mid-infrared (MIR) signal emitted from an object is proportional to the surface emissivity (*ε*) and the fourth power of its absolute temperature (*T*). For against the passive detection of thermal targets [7], low emittance in the working band is a valid approach to realize thermal camouflage. With the advancement of IR lidars, it makes possible to actively detect and identify infrared targets, likes the microwave radars. For avoiding these IR lidars [8], high absorption in the detection wavelength is a coveted approach to achieve infrared laser stealth. At the temperature below the 1000 °C, the potential of MIR radiation heat transfer far exceeds the half of the total blackbody radiation of the heat dissipation [9]. Therefore, MIR radiation thermal management is a potential approach for cooling the hot object without extra energy. With the strong demand from personal heat management, energy utilization, and military security [10–12], some researchers pay more attention to the infrared wavelength-selective emitters.

Earlier, using some metal materials with low emissivity is a viable method against thermal detection [13–15]. The entirely low emissivity in the dual-band MIR of mid-wavelength-infrared (MWIR, 3–5 μm) and long-wavelength-infrared (LWIR, 8–14 μm) still is a challenge to realize laser stealth (such as CO_2_ laser at 10.6 μm). In order to face the challenges in the MIR camouflage, Pan et al. [16] realized infrared camouflage compatible with thermal management using the metamaterials, which can cope with dual-window thermal camouflage, thermal management and infrared laser stealth. High aspect infrared selective emitter designs are desirable in the multi-functional infrared camouflage [17]. However, the compatibility between the MIR camouflage and near-infrared (NIR) stealth still have some troublesome. A limiting factor in the development of IR camouflage with thermal management is the lack of delicate design that are suitable to dispersion of optics material, variation of optics loss, and easy to machine.

Hierarchical metamaterials, an artificial structure in which the point-to-point design of structures and wavelengths in a large working wavelength, are well applied to multispectral camouflage [18–20]. Indeed, light–matter interactions are inherently hierarchical – the strong interference effects of thin film rely on the working wavelength and complex refractive index. Especially, one-dimensional photonic crystals which are often made by dielectric film with quarter-wavelength thickness, such as Fabry−Pérot (FP) cavities [21], distributed Bragg gratings [22] and hyperbolic metamaterials [23], brought the extraordinary optical phenomena and the wide application prospects. On the one hand, a small change of thickness can cause a strong peak shift at short waveband. On the other hand, such a small change of thickness cannot cause a strong peak shift at long waveband. Introducing the principle of hierarchical metamaterials in the multilayer film structures (MFSs) not only have the potential for multispectral compatibility, but also avoid the complicated fabrication of metamaterial processing.

In our previous works, combining the inverse design [24] and the ideal selective emission spectrum, the selective emitters have been designed and implemented for solar absorption, radiative cooling, and tunable thermal stealth [25–27]. Here, we experimentally demonstrate a high-performance selective MFS emitter implementing on infrared camouflage with thermal management. The MFS consists of five materials (SiO_2_, Ge, ZnS, Pt, and Au) for 7-layer thin-film stacking structure with the thickness of 3.735 μm. The inverse design of genetic algorithm (GA) has been used for designing the MIR part of the spectrum which demands to realize high reflectance of dual-band MIR atmospheric windows (MWIR and LWIR), high emittance in the nonatmospheric window (5–8 μm) and high absorbance for 10.6-μm CO_2_ laser. In the part of the spectrum, the optimization which inspired by the hierarchical metamaterial has been used to NIR laser stealth. Both of infrared camouflage performance and thermal management performance are measured by experiment. The experimental and theoretical results are in good agreement. Moreover, the polarization-independent and angular-independent selective emission of the MFS has been verified by experimental and calculated results. Besides, the proposed MFS is fabricated using inexpensive, layer-by-layer deposition of E-beam evaporation technique; this enables us to fabricate MFS with a large area (six-inch silicon wafers), which is limited only by the scale of warehouse. The high performance of infrared camouflage with thermal management based on inverse design and hierarchical metamaterial and the potentials for lager area processing make it suitable for industrial radiative cooling and military application of concealment.

## 2 Methods

### 2.1 Principle of infrared camouflage with thermal management

**Figure 1:**
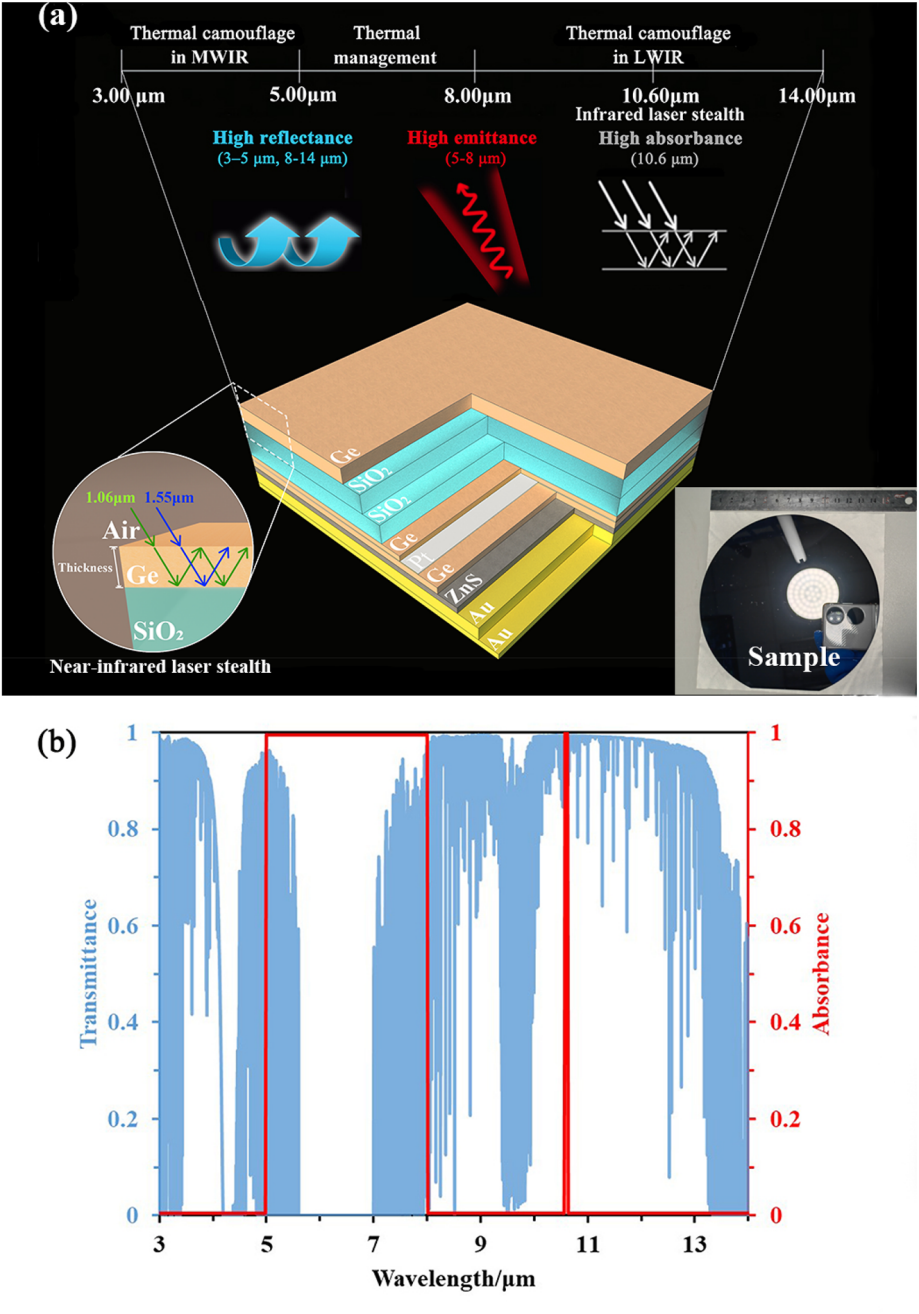
Inverse design and hierarchical metamaterial for IR camouflage with thermal management. (a) Application scenarios for the thermal camouflage in the dual-band infrared transparent window, thermal management in non-window band, and laser stealth in NIR band (1.06 μm and 1.55 μm) and MIR band (10.6 μm) based on the MFS of inverse design and hierarchical metamaterials. The image on the bottom right shows a device fabricated on a 6-inch silicon wafer. (b) Transmittance spectrum in the dual-band infrared transparent windows and ideal MIR spectral characteristics.

The concept of implementing the infrared camouflage with thermal management and the application scenarios of MFSs which combining inverse design and hierarchical metamaterial are shown in Figure 1(a). In the concept, infrared camouflage including the thermal camouflage in the dual-band MIR transparent windows and infrared laser stealth within the band of 1.06 μm, 1.55 μm, and 10.6 μm has been considered. The large-area sample is fabricated on a 6-inch silicon wafer. The proposed MFS is able to realize thermal camouflage, thermal management and infrared laser stealth within the NIR band and the MIR band.

The transmission of dual-band MIR transparent windows (MWIR and LWIR) is marked by the blue line in Figure 1(b). Because of the high transmission of dual-band MIR transparent windows, most of detections are working in these bands. Thermal camouflage is mainly aimed to conceal under the MIR detections. Low emissivity (high reflectance) is a crucial standard for thermal camouflage. Thermal management mainly realizes energy dissipation through thermal radiation in the non-atmospheric window (5–8 μm), and this part of energy dissipation is difficult to be detected by detection equipment. Considering the development of MIR lidar, the 10.6-μm-laser in the long-wave infrared window is often used for laser navigation. Therefore, the proposed MFS suppresses this part of infrared information by absorbing the laser signal. Based on the above characteristics [28, 29], the ideal MIR spectral characteristics are shown by red line in Figure 1(b).

Besides, the principle of hierarchical metamaterials has been used to realize the laser stealth in the NIR band. By regulating thickness of the MFSs top layer, the MFS has been optimized to achieve strong dual-band absorption near the NIR band of 1.06 μm and 1.55 μm.

### 2.2 Design of GA

The MFS based on inverse design has been applied for selective emitter. Most of them [30–32] are realized by using transparent dielectric materials in the tailored bands. Some of these studies [22, 33] use pre-determined high and low refractive index layers to optimize distributed Bragg reflector (DBR). In order to satisfy Bragg interference conditions and produce photonic band gaps, a considerable amount of high and low refractive index stacked layers are required. In addition, the original needle optimization method, which is more oriented to the evaluation function by continuously introducing abrupt refractive indices, leads to a further increase in the number of layers. However, the MFSs based on the DBR and the needle optimization are too complex to fabricate. The inverse design of MFSs based on multi-material systems promises to explore more possibilities of material combinations, few layers of the thin films, and better spectral characteristics consistent with the evaluation function.

Here, eight materials which include five transparent materials in the infrared band (SiO_2_, Ge, ZnS, Si, and Ge_2_Sb_2_Te_5_) and three metals (Au, Pt, and Ag) are in the material database. Au is preset as the substrate to have lower emissivity in the initial stage. The inverse design method is realized by combining ideal infrared camouflage spectral properties and GA to find MFSs with higher performance. Here, the figure of merit (FOM) is defined as,

(1) $$\begin{array}{ll} FOM_{1} & =0.25\times R_{1}+0.25\times \left(1-R_{2}\right)\\ & \;+0.25\times R_{3}+0.25\times \left(1-R_{4}\right). \end{array}$$

**Figure 2:**
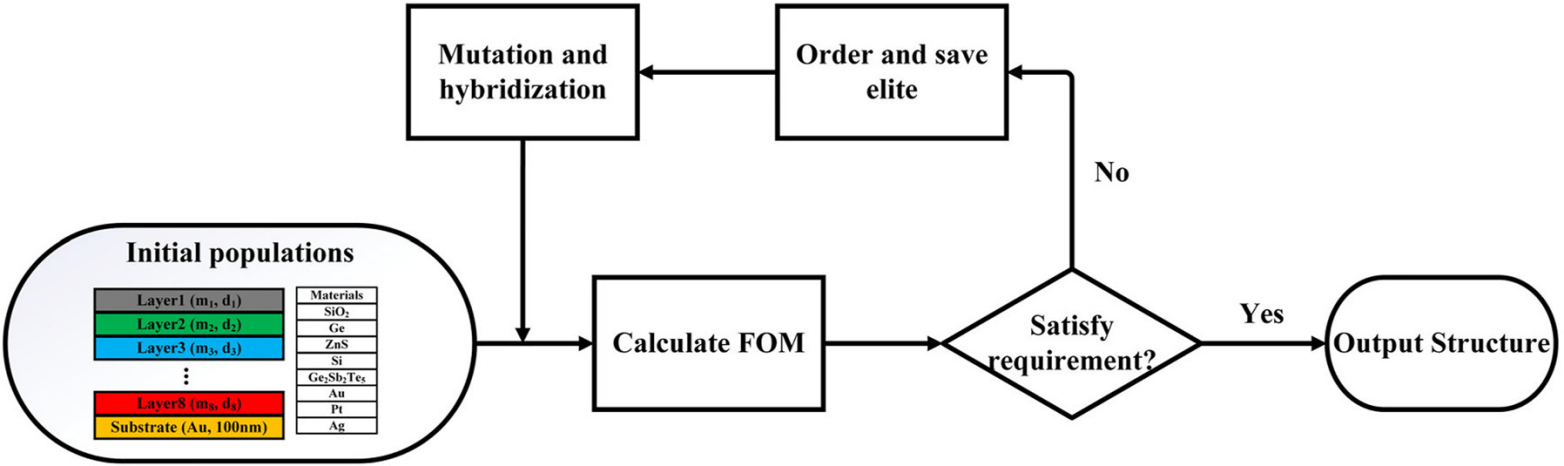
Designed schematic of the GA optimization. The steps of the GA optimization design include initial populations, calculating FOM, ordering (save elite), mutation and hybridization, and outputting the optimal MFS.

Here, *R*_1_, *R*_2_, *R*_3_, and *R*_4_ stand for the average reflectance of 3–5 μm, 5–8 μm, 8–14 μm, and 10.6 μm, respectively. Owing to the similarity between FOM and ideal MIR spectral characteristics, FOM_1_ was adopted as a mid-infrared compatible efficiency evaluation standard in the MIR band. During the inverse design process, we considered an 8-layer thin film structure with the substrate of Au (100 nm). Each of the inverse-design layers are the thickness between 0 and 1000 nm. The details of GA are shown in Figure 2. Our implementation of the memetic evolutionary algorithm consists of the generation of initial populations, calculating FOM, ordering (save elite), mutation, hybridization, and outputting the optimal MFS. The specific settings of the GA have been reported in the previous works [24, 25]. The initial population is composed of 1000 individuals, which are randomly combined by the 8 materials (SiO_2_, Ge, ZnS, Si, Ge_2_Sb_2_Te_5_, Au, Pt, and Ag) and the thickness (0 ∼ 1000 nm) of 8 layers. In the GA, these individuals, which are defined by 8 binary numbers with numbering (material information) of 10 bits, represent the 8-layer MFS. In the process of GA, population size is 1000, the mutation rate is 30 %, the hybridization rate is 75 %, and the number of iterations set 50 generations.

Combining the FDTD methods and GA, it takes about 60 h to get the MFS on a computer with a 6-core central processing unit (Intel Core i7-9750H). The total memory of the computer is 32 GB (SAMSUNG DDR4 2666 MHz). After completing the whole optimization process, an inverse-design MFS is obtained, and the parameters are listed in Table 1. Wherein some of the layers showed degradation including the layer merging (layer 2 and layer 3) and layer repetition (layer 8). The degradation reduced the number of layers and benefitted the fabrication. Due to the selection of GA, three materials, Ag, Si, and Ge_2_Sb_2_Te_5_, were eliminated during the iteration of the genetic algorithm, which is related to the dispersion of the materials in the MIR spectral range.

**Table 1:** Material and thickness setup assigned for each layer of the proposed MFS after GA optimization process.

Layer	Substrate	1	2	3	4	5	6	7	8
Material	Au	Ge	SiO_2_	SiO_2_	Ge	Pt	Ge	ZnS	Au
Thickness (nm)	100	763	962	969	276	40	349	304	645

**Figure 3:**
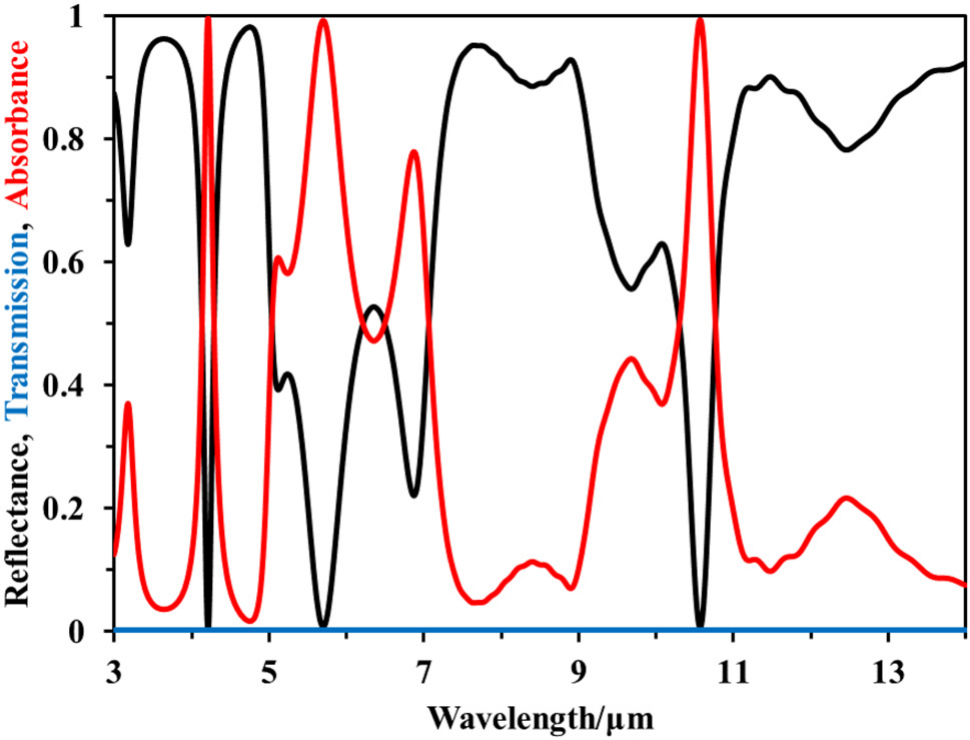
Calculated spectral reflectance (black line), transmission (blue line), and absorbance (red line) of the inverse-design MFS in the MIR band.

In Figure 3, we give the MIR spectra of the inverse engineered MFS. Among them, red line, black line, and blue line represent absorbance (emissivity), reflectance, and transmittance, respectively; wherein the absorbance is derived from reflectance and transmittance. The calculated results demonstrated the high performance of thermal camouflage (*R*_3–5μm_ = 0.82, *R*_8–14μm_ = 0.77), infrared laser camouflage (*ε*_10.6μm_ = 98 %) and thermal management (*ε*_5–8μm_ = 67 %). According to the Eq. (1), the proposed MFS has the high compatible efficiency (FOM_1_ = 81 %).

### 2.3 Optimization of hierarchical metamaterial

Hierarchical metamaterials, an artificial structure in which the point-to-point design of structures and wavelengths in a large working wavelength, are well applied to multispectral camouflage. The wavelength difference between the NIR band and the MIR leads to the fact that a weak change of thin films’ thickness has a much greater effect on the NIR than on the MIR band.

**Figure 4:**
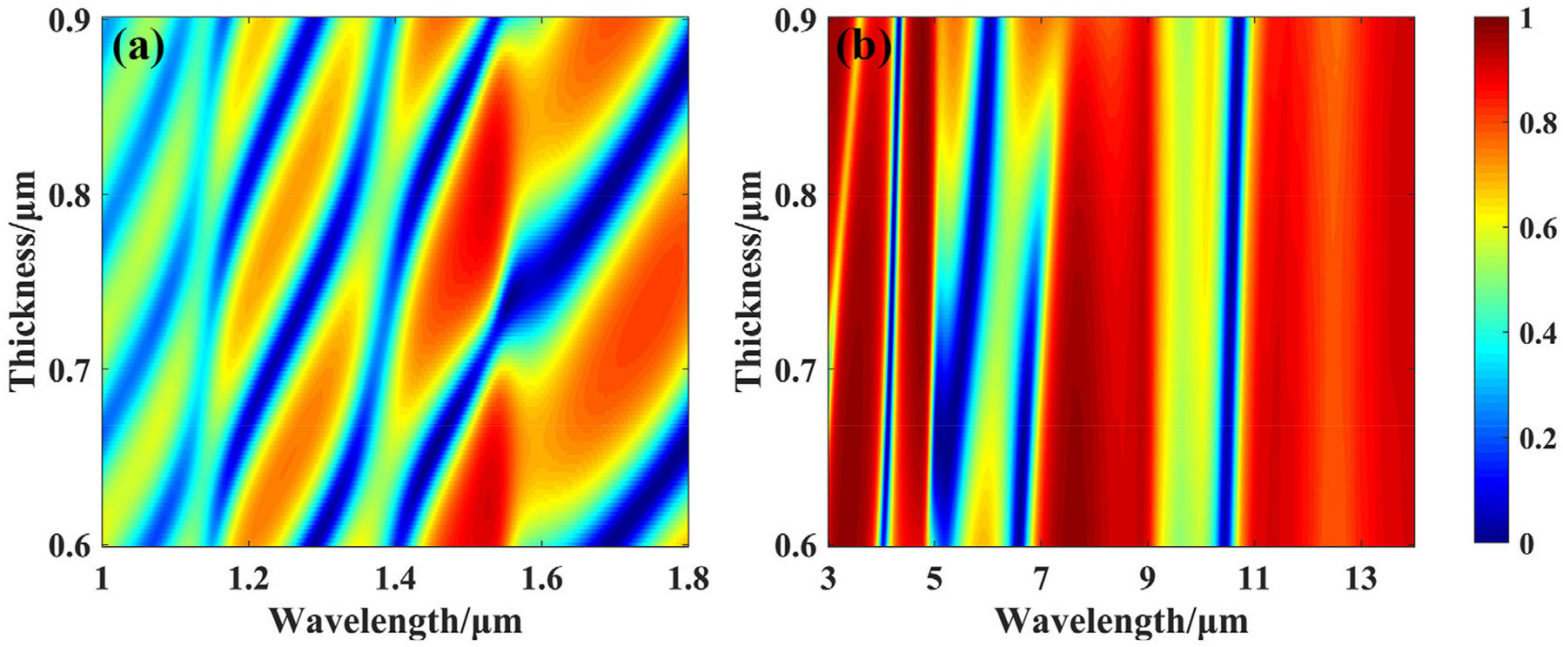
Calculated spectral reflectance of the optimized MFS with the top-layer thickness increase from 600 nm to 900 nm in the NIR band (a) and MIR band (b).

Inspired by the hierarchical metamaterials, we achieved compatibility with NIR laser stealth by optimizing the tiny thickness of the inverse-design MFS. Interestingly, the absorption properties in the NIR are mainly derived from the asymmetric FP cavity effect between Ge layer and metals. The materials of SiO_2_ and ZnS can still be considered transparent in the NIR. As a result, the eigen dispersion of materials can be associated with hierarchical metamaterials. Under the inverse-design MFS of GA, we demonstrated this by tuning the thickness of the top Ge layer from 600 nm to 900 nm, which shows that the characteristic of hierarchical metamaterials between NIR and MIR in Figure 4(a) and (b). The origin thickness (763 nm) of top Ge layer which has been generated by the GA design is included in the regulation range (600 nm–900 nm) of the hierarchical optimization.

On the one hand, a small change of thickness can cause a strong peak shift at short waveband. In the NIR band, the thickness change (∼100 nm) of Ge layer can realize the switch of accumulating phase. For the wavelength of 1.06 μm, the absorption peak at the thickness of 720 nm and the absorption valley at the thickness of 780 nm. For the wavelength of 1.55 μm, the absorption peak at the thickness of 740 nm and the absorption valley at the thickness of 840 nm. On the other hand, such a small change of thickness cannot cause a strong peak shift at long waveband. In the MIR band, the 100 nm-thickness change of Ge layer will not bring about strong absorption peak drift. For the wavelength of 10.6 μm, the absorption is mostly maintained above 90 % at thicknesses ranging from 700 nm to 900 nm.

**Figure 5:**
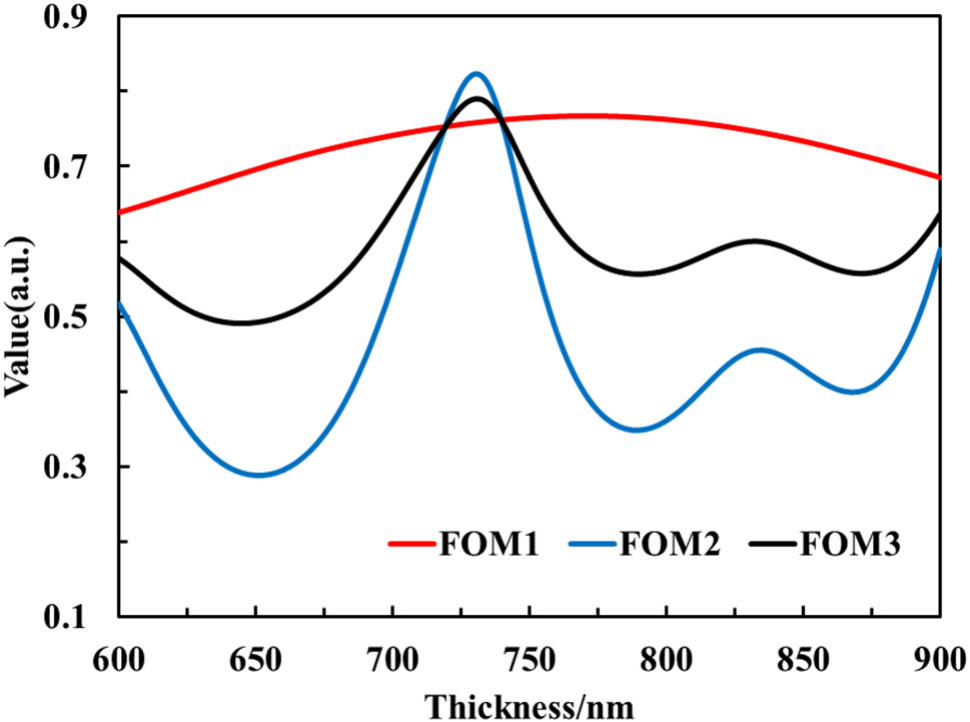
The FOM comparisons including FOM_1_ (red curve), FOM_2_ (blue curve), and FOM_3_ (black curve) with the top layer thickness increasing from 600 nm to 900 nm.

In order to implement dual-band absorption in the NIR and stealth compatibility between the NIR band and MIR band, we establish a NIR dual-band optimization (FOM_*2*_) and a joint optimization function (FOM_3_) for the IR multi-wavelength band,

(2) $$FOM_{2}=0.5\times A_{1.06\mu m}+0.5\times A_{1.55\mu m},$$

(3) $$FOM_{3}=0.5\times FOM_{1}+0.5\times FOM_{2}.$$

where *A*_1.06μm_ and *A*_1.55μm_ represent the absorbances at the wavelength of 1.06 μm and 1.55 μm, respectively. As shown in the Figure 5, we give the NIR dual-band optimization curves with thickness and the variation curves of the joint optimization. Finally, the optimized top Ge thickness is 735 nm.

### 2.4 Fabrication of devices

The devices were prepared using electron beam evaporation deposition on a 6-inch silicon wafer. During the preparation process, the vacuum of the chamber was about 6 × 10^−3^ Pa, the annealing temperature was about 50 °C. The deposition rate of Ge, SiO_2_, Pt, and Au were about 1 nm/s and the deposition rate of ZnS was about 2 nm/s, respectively, which were monitored by a quartz crystal oscillator.

### 2.5 Measurement of devices

The devices were optically characterized using FTIR (Nicolet Continuum) and NIR Spectrophotometer (HITACHI U4100) to obtain the experimental reflectance spectra of NIR and MIR, respectively. The working wavelength of the infrared camera is from 8 μm to 14 μm. A Hitachi S-4800 field emission scanning electron microscope (FESEM) was employed to observe the MFS infrared camouflage.

## 3 Results and discussion

### 3.1 Optical measurement and physical mechanism

**Figure 6:**
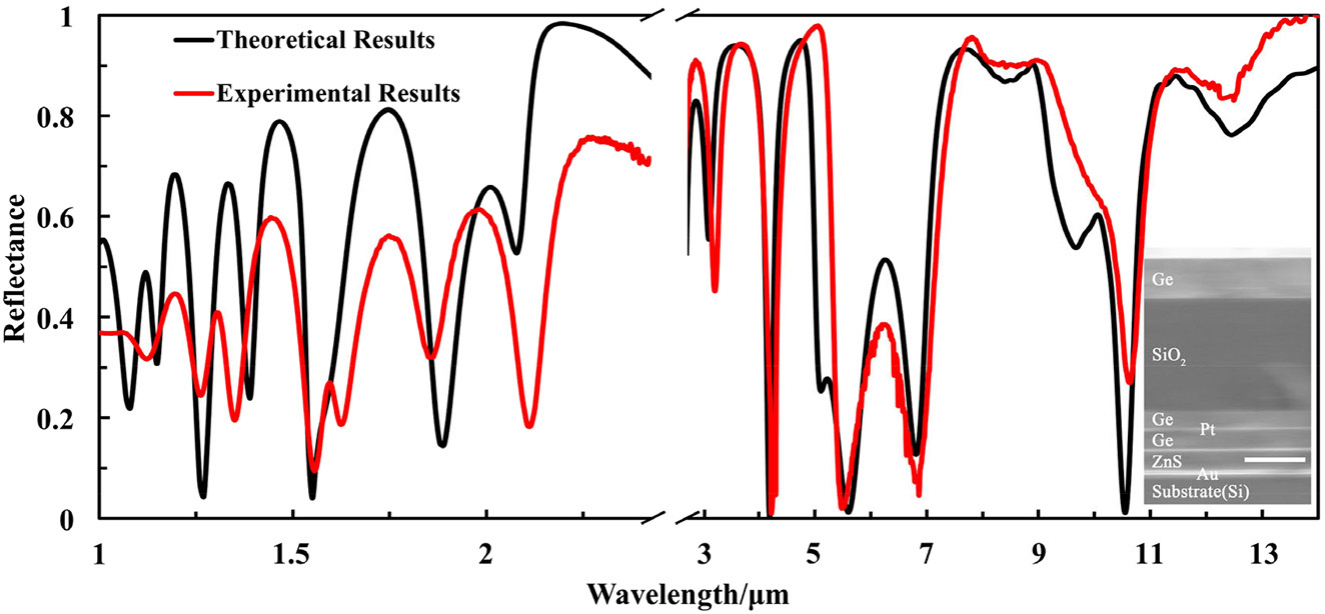
The measured reflectance (red lines) of the 3.735-μm-thick MFS from 1 μm to 14 μm. Theoretical results for the same hybrid metamaterial structure (black lines) are plotted for comparison. The thicknesses of the MFS are 0.735/1.931/0.276/0.04/0.349/0.304/0.1 μm (from top to bottom). The inset shows the FESEM image of the fabricated MFS (seven layers with substrate), with a scale bar of 1 μm.

For normal incidence, the experimental results and the theoretical results show excellent agreement in the Figure 6. The experimental results illustrate that the thermal camouflage with high reflectance in dual-band MIR transparent windows (*ε*_3–5μm_ = 0.21, *ε*_8–14μm_ = 0.16), the laser stealth in both NIR and MIR band (*ε*_1.06μm_ = 0.64, *ε*_1.55μm_ = 0.90, *ε*_10.6μm_ = 0.76), and thermal management in the non-atmospheric window (*ε*_5–8μm_ = 0.54). The slight differences between experiments and simulations are attributed to errors in the film thickness and the addition of a buffer layer between the Ge and SiO_2_ layers.

**Figure 7:**
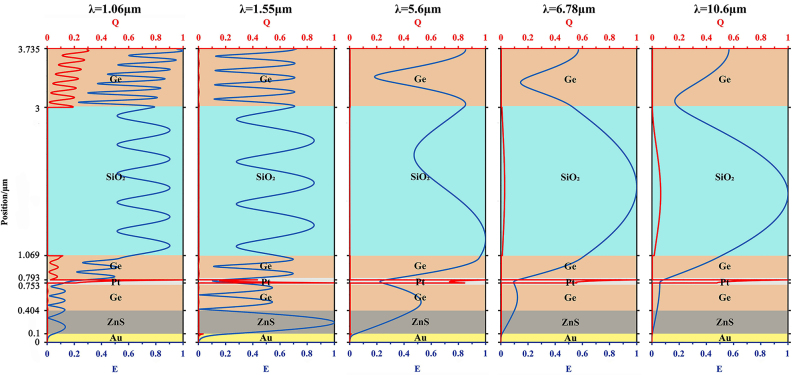
Profile of normalized electric field intensity and loss distribution of the proposed MFS in the 1.06 µm, 1.55 µm, 5.6 µm, 6.78 µm, and 10.6 µm.

In order to study the physical mechanism of the selective emitter, the electric field distribution for the absorption peaks, including the wavelength of 1.06 μm, 1.55 μm, 5.6 μm, 6.78 μm, and 10.6 μm are shown in Figure 7 with the blue line. It can be seen that, in the NIR band, the optical absorption is mainly attributed to the low-loss medium (Ge) and metal reflective layer introduced in the asymmetric FP resonance [34–36]. In contrast, the absorption in the non-atmospheric window mainly arises from the evanescent wave at the metallic Pt, which would be related to the skinning depth of the metal [37]. As the wavelength extends into the LWIR, SiO_2_ gradually changes its optical properties from a transparent medium to a low-loss medium. The absorption peaks in the wavelength of 6.78 μm and 10.6 μm can be attributed to asymmetric FP resonance between the SiO_2_ layer and metal reflective layer. According to the ohmic loss theory, the loss distribution of the proposed MFS is plotted by the red line in Figure 7. As the main body of loss, metal plays an important role in the absorption of multi-wavelength light waves. Wherein, the contribution of Pt metal reflective layer to absorption is 12 %, 78 %, 89 %, 39 %, and 25 % corresponding to wavelength of 1.06 μm, 1.55 μm, 5.6 μm, 6.78 μm, and 10.6 μm, respectively. The remaining energy is mainly dissipated by the opaque material (Ge layer and SiO_2_ layer) in these bands.

### 3.2 Infrared camouflage performance

**Figure 8:**
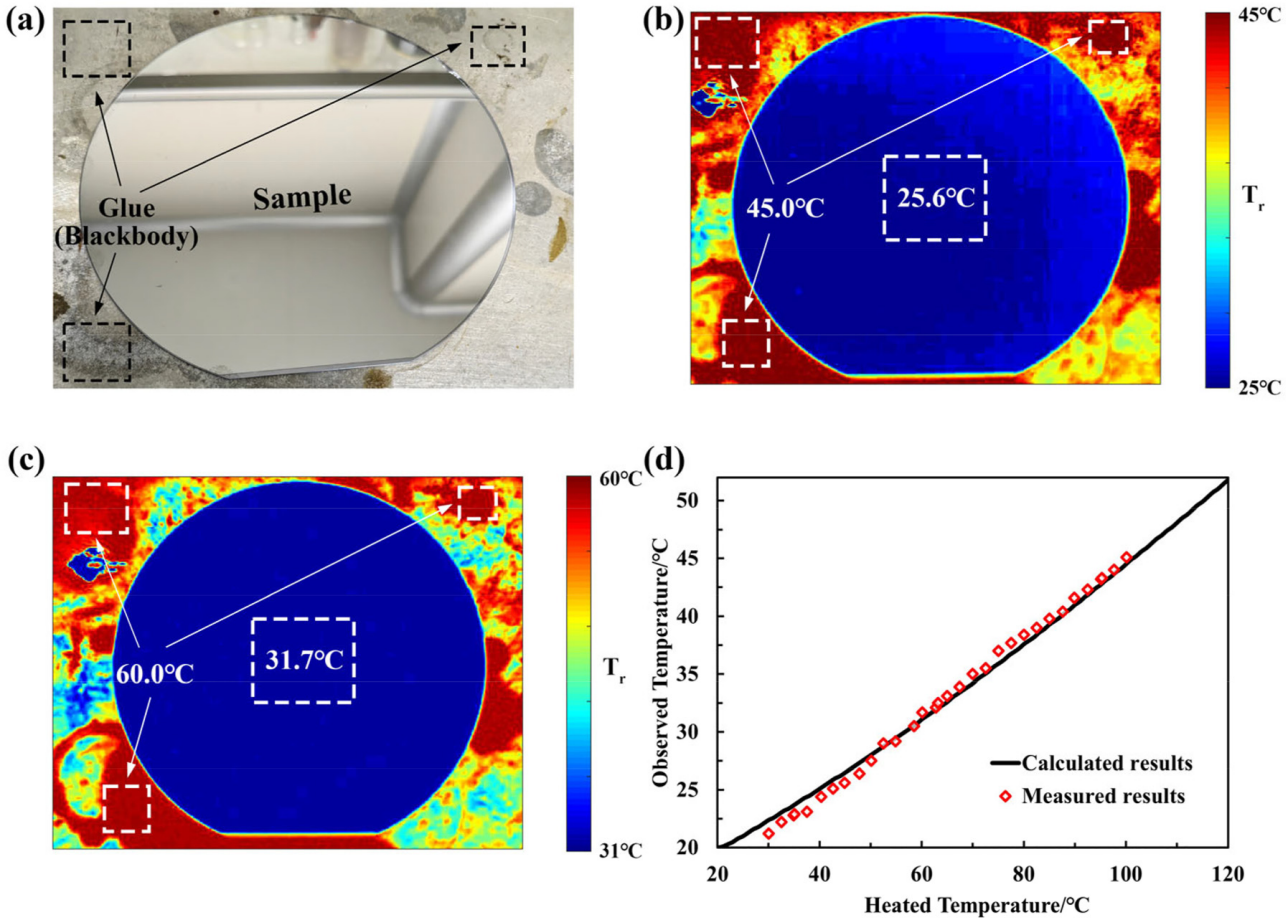
Demonstrations of IR camouflage MFS and observed temperature regulation. (a) Image of the sample of the MFS and the heating table with glue. (b) IR image of the MFS heated at 45 °C with the background temperature of 20 °C. (c) IR image of the MFS heated at 60 °C with the background temperature of 20 °C. (d) The relation between observed (radiation) temperatures and heated (real) temperatures of the sample.

As shown in Figure 8(a), we performed thermal imaging of the experimental sample indoors using a heating table coated with a glue, where the emissivity of the glue is close to that of a blackbody in the LWIR and used to accurately calibrate the heating temperature. In Figure 8(b) and (c), the proposed devices, heated at 45 °C and 60 °C, exhibit the observed temperature of 25.6 °C and 31.7 °C, respectively. The observed temperature is well below the heating temperature and convictive to illustrate the thermal camouflage performance of the proposed devices.

Interestingly, according to the Stefan–Boltzmann law, the temperature of object can be solved by detecting the radiation power with specific band [16, 21, 38],

(4) $$T_{r}=P^{-1}\left(\varepsilon_{IR},T\right),$$

here, *T*_*r*_ is the temperature obtained by the inverse function of the blackbody radiation spectrum. *P* is the radiation power detected by the IR detector, which corresponds to the radiation power radiated by the black body at temperature *T*. The *ε*_IR_ is the emissivity of the IR detector in the work wavelength (8–14 μm), for the infrared camera is *ε*_IR_ = 1. The detection power of infrared imaging is generally determined by emitted radiation from object and the reflection radiation from environment,

(5) $$\begin{array}{ll} P\left(\varepsilon ,T\right) & =P_{\text{rad}}\left(\varepsilon ,T\right)+P_{\text{ref}}\left(\varepsilon ,\varepsilon_{a},T_{a}\right)\\ & =\varepsilon \left(\lambda \right)I_{BB}\left(T\right)+\left[1-\varepsilon \left(\lambda \right)\right]\varepsilon_{a}\left(\lambda \right)I_{BB}\left(T_{a}\right). \end{array}$$

Among them, *ε* and *ε*_*a*_ are the emissivity of the object and ambient environment, respectively. *T* and *T*_*a*_ represent the temperature of the object and the temperature of the ambient environment, respectively. *I*_BB_ represents the blackbody irradiance at the corresponding temperature, and the blackbody radiation threshold is determined by the working wavelength of the infrared camera. Both of the calculated and measured results from heat temperature from 20 °C to 120 °C of the device have been plotted in the Figure 8(d). These results further illustrate the important role of the low emissivity (high reflectivity) of the transparent window region for thermal camouflage.

### 3.3 Thermal management of radiative cooling performance

**Figure 9:**
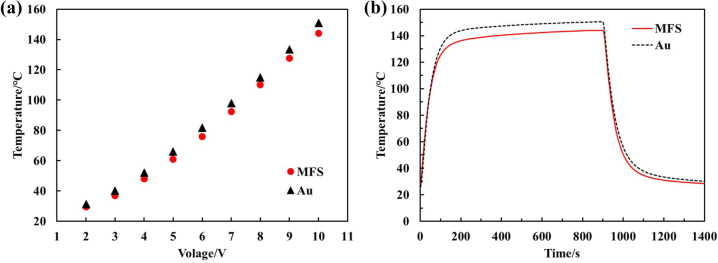
The thermal measurement of the MFS emitter. (a) The steady-state temperatures of the MFS emitter and equal-sized Au film heat with the different heating voltage. (b) Real temperature variations of the MFS emitter and Au film samples with the same heating power (with the voltage of 10 V) during thermal measurements in the practical environments.

Recently, passive radiative cooling has been widely studied as one of the ways of thermal management [39–41]. In particular, radiation transfer in the non-atmospheric window region will provide the possibility of thermal camouflage for compatible thermal management by metamaterials [14, 16, 22]. The thermal management by radiative cooling is characterized a lower steady-state temperature and shorter cooling time of the hot object in this work. Comparing with the conventional metal film of Au, the thermal management performance of the proposed MFS emitter is verified in the practical environment. The experimental device which considered to minimizing the convection heat transfer and conduction heat transfer was depicted in the Figure S1. We compare the steady-state temperatures of the two samples of the MFS film and equal-sized Au films under different heating voltages in the Figure 9(a). The results illustrate that the MFS film exhibits a lower steady-state temperature than the Au film at the same voltage. At the voltage of 10 V, the highest steady-state temperature difference between the two films reaches 7 °C. As shown in Figure 9(b), we compare the temperature of two samples versus time under the same heating power of 10 V for 15 min. The temperature and time are recorded by the thermocouple from the room temperature to the stable-state heating temperature and then back to the room temperature by the natural cooling. It can be seen that the steady-state temperature of the MFS emitter is 144 °C, while the steady-state temperature of the metal film is 151 °C. During natural cooling, the MFS emitter takes 144 s to reduce from 144 to 40 °C, while the Au film device takes 170 s to complete the same temperature change. The experimental results illustrate the significances of radiant heat management for heat dissipation from high temperature objects.

**Figure 10:**
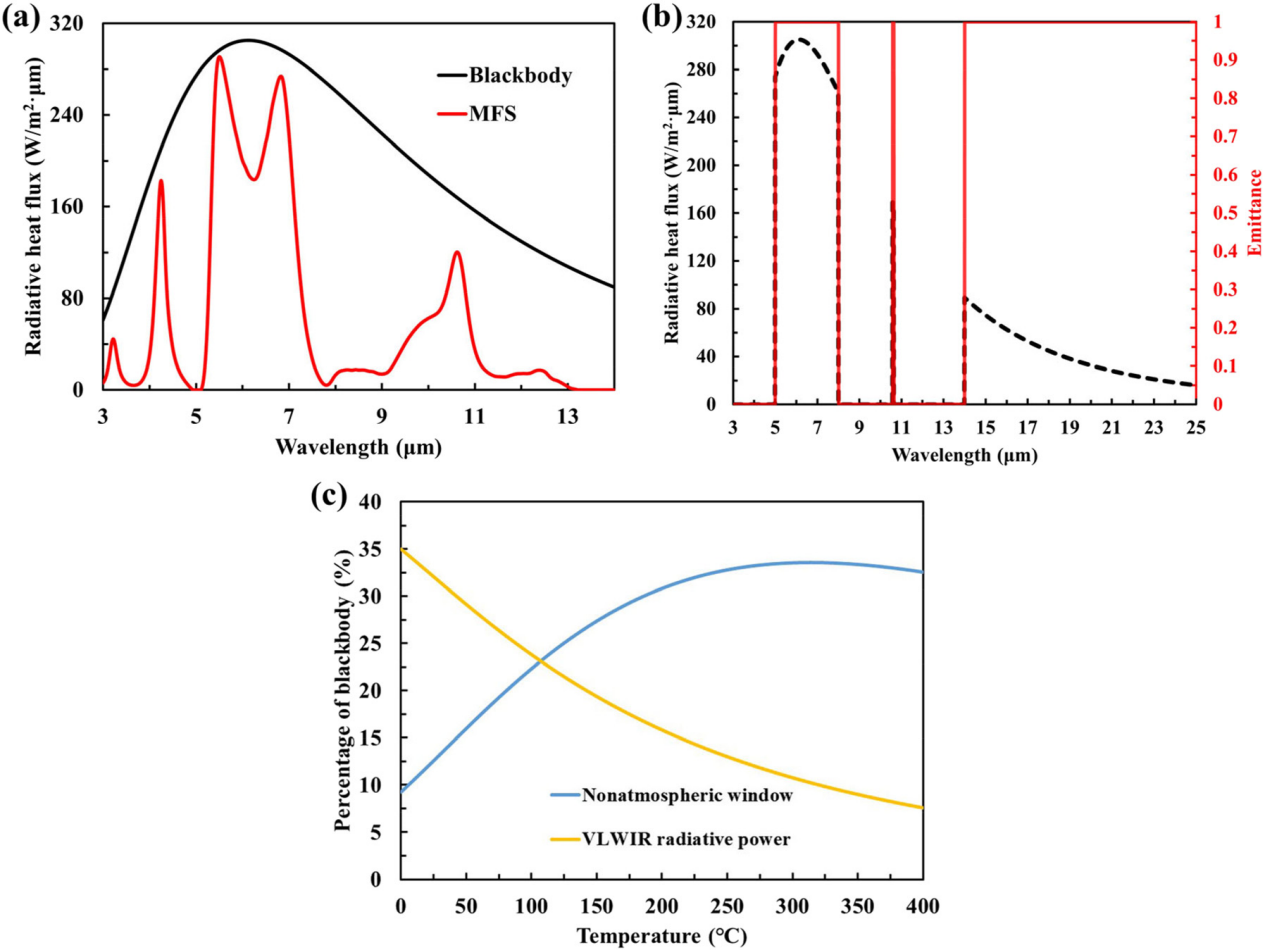
Themal management of radiative cooling from non-atmospheric window to VLWIR band. (a) The calculated radiative heat flux of the blackbody and MSE at 473 K, the black and red lines represent the blackbody and the MFS. (b) The ideal selective emitter extends the thermal management to the VLWIR band and realize IR camouflage. The radiation power of the ideal selective emitter with the temperature of 200 °C is plotted by the black dash line. The idea spectral characteristics from MWIR band to VLWIR band are plotted by the red line. (c) The share of non-atmospheric-window radiative power and VLWIR radiative power in the total energy of blackbody radiation.

The thermal management performance of the proposed MFS emitter has also been calculated in the Figure 10(a). The blackbody radiation properties of the ideal blackbody and the proposed MFS at the temperature of 200 °C have been plotted with the black and red lines, respectively. The calculated integral thermal radiation power of the proposed MFS is about 695 W/m^2^. Because of the platform-like absorption in the wavelength of 10.6 μm for the laser stealth, the proposed MFS emitter still has thermal management in the LWIR band, which accounts for about 24 % of the total integrated thermal radiation power. Interestingly, previous studies of thermal camouflage with thermal management have not considered the contribution of blackbody radiation in the very long-wavelength infrared (VLWIR) for thermal camouflage with thermal management. As shown in Figure 10(b), we extended the ideal spectral profile to the VLWIR in order to further enhance the thermal management performance. Compared to the mid-infrared ideal model (Figure 1(b)), the ideal spectral profile to the VLWIR band (14–25 μm) is improved in thermal management performance from 876 W/m^2^ to 1326 W/m^2^ with the temperature of 200 °C. According to the Planck’s law and the Stefan–Boltzmann law, we give the percentage of wavelength-band emission (non-atmospheric window, 5–8 μm; VLWIR, 14–25 μm) relative to blackbody radiation (*ε* = 1) for ideally selected emitters at different temperatures. As shown in Figure 10(c), energy dissipation is achieved by compatible non-window selective emission and VLWIR emission with more than 40 % of the blackbody’s radiative heat power. At room temperature (0–40 °C), VLWIR radiative thermal management is superior to non-atmospheric window radiative thermal management. As the temperature increases, non-atmospheric window radiative thermal management gradually dominates.

In Table 2, we draw a comparison between our results and the recent work of infrared camouflage with thermal management. The infrared laser stealth needs the high absorption in the work band of the infrared detector including the 1.06 μm, 1.55 μm, and 10.6 μm. The thermal camouflage requires low emittance to allow the hot object to be hidden from the background environment. The average emittances in the dual-band infrared transparent windows are discussed. The thermal management can realize the energy dissipation in some undectable infrared bands likes the blackbody. We main consider the non-atmospheric-window radiative power as the performance of the thermal management. Interestingly, the VLWIR radiative cooling which has been discussed above also can be considered in the future thermal management.

**Table 2:** Comparison of the performance for infrared camouflage with thermal management.

Ref.	Materials	Structure	Thermal camouflage		Infrared laser stealth			Thermal management
			(*ε*_3–5 μm_, *ε*_8–14 μm_)		(*ε*_1.06 μm_, *ε*_1.55 μm_, *ε*_10.6 μm_)			(*ε*_5–8 μm_)
[14]	Ag/Ge	Thin films	0.18	0.31	×	×	×	0.82
[16]	Si/GST/Au	Gratings	0.25	0.33	×	×	0.9	0.77
[27]	GST/Au	Gratings	0.17	0.16	×	×	×	0.85
[29]	ZnS/Ge/SiO_2_	Thin films	0.11	0.12	×	0.9	0.9	0.61
[42]	Al/Ge/Ag	Gratings	0.15	0.03	0.9	×	×	×
MFS	SiO_2_/Ge/ZnS/Pt/Au	Thin films	0.21	0.16	0.64	0.90	0.76	0.54

As shown in Table S1, we compare the geometric parameter, processing methods, and spectral control properties of different 1D (multilayer films), 2D, and 3D metamaterials. In general, MFSs achieve coherent absorption through layer-induced phase accumulation. MFSs require more careful thickness calculations and material combinations to further improve the emission efficiency of wavelength-selective emitters. Two- and three-dimensional metamaterials achieve optical field modulation in higher dimensions and thus tend to have more precise spectral control. Most of them have high average absorption (>85 %) in the working band. However, the combination of broadband and narrowband emission needs to be further explored to be compatible with the needs of different functional and application scenarios. In terms of the size of the prepared devices, MFSs are usually suitable for large-area fabrication due to the use of existing material researches and mature fabrication processes. In contrast, 3D printing and laser direct writing techniques are potential methods to achieve large area preparation for 2D and 3D metamaterials [11, 43, 44]. Interestingly, it is expected to achieve multi-spectral complementarity by combining multiple dimensions metamaterial configurations inspired to the hierarchical metamaterials.

### 3.4 Angle independence of the MFS emitter

**Figure 11:**
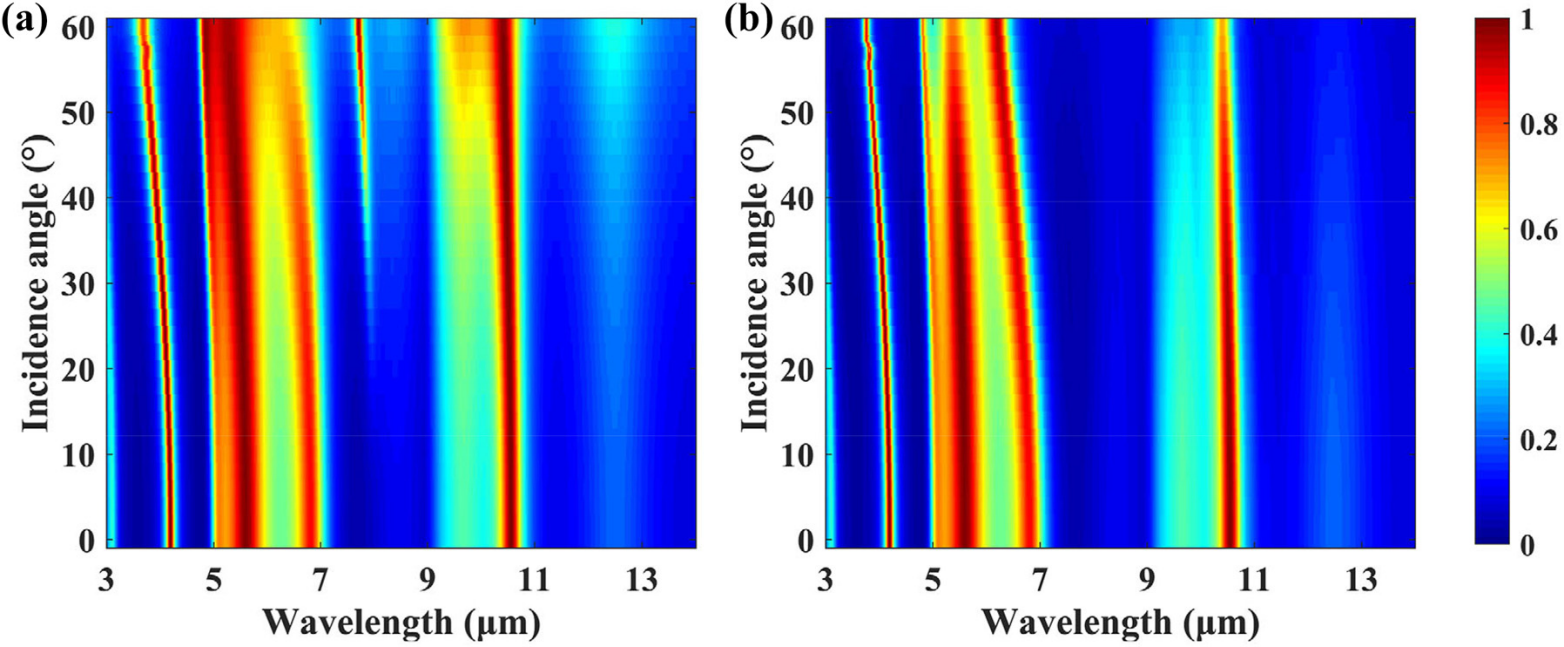
The calculated emissivity/absorptivity of p-polarized (a) and s-polarized lights (b) as a function of the incident angle for the proposed MFS.

As shown in Figure 11(a) and (b), the designed MFS is iridescence-free from normal incidence to an incident angle up to 60°. Both the p-polarized light wave and the s-polarized light wave support large angular incidence with the 60°. This is due to the high refractive index of Ge [45] and the metal reflective layer of Au and Pt [46]. Moreover, the experimental reflectance versus wavelength and incident angle for unpolarized NIR and MIR light are shown in Figures S2 and S3. The experimental results agree with the calculated results and illustrate the robust incidence angle independence of the proposed MFS. The angular independent performance for unpolarized light incidence which approaches the application scenarios is an important evaluation indicator for thermal camouflage and infrared laser stealth.

## 4 Conclusions

The synergy between the inverse design methods and hierarchical metamaterial opens up a new paradigm to address multi-material, multi-spectral compatible, and wavelength-selective problems. Here, we proposed a new platform using an MFS with multiple interference and hierarchical metamaterial to realize infrared camouflage with thermal management over a wide spectral range. We experimentally demonstrate the performance of angular-independent MFS including NIR and MIR laser stealth, thermal camouflage, and thermal management. NIR dual-band laser stealth is achieved by hierarchical design of low-loss films (Ge) with metal absorption effects. The absorption peaks of 1.06 μm and 1.55 μm which effectively avoid the reflected laser signal detectors are greater than 64 % and 90 %, respectively. The inverse design is used to realize multifunctional compatibility among thermal management, thermal camouflage, and MIR laser stealth. The thermal imaging agrees with the calculated results and illustrates that the MFS is able to achieve thermal camouflage by making the hot object look as cold as the background temperature. The thermal management properties are experimentally demonstrated to have significant radiation heat dissipation performance compared to conventional metals. Moreover, we extend the concept of VLWIR thermal management based on the non-atmospheric window thermal management, which will investigate the potential applications of thermal management properties at room temperature or higher temperatures. Therefore, these attractive properties of MFS selective emitter suggest promising broad applicability in many fields related to heat management, energy utilization, and military security.

## Supplementary Material

Supplementary Material Details
