# Supplementary material for: Implementing of infrared camouflage with thermal management based on inverse design and hierarchical metamaterial
Source: Nanophotonics. 2023 Apr 13;12(10):1891–902. doi: 10.1515/nanoph-2023-0067 (PMC11501626; doi:10.1515/nanoph-2023-0067)
Supplement: Supplementary file 1 — Supplementary Material Details [file j_nanoph-2023-0067_suppl_001.pdf]

Supplementary material

Implementing of Infrared Camouflage with Thermal Management Based on Inverse Design and Hierarchical Metamaterial

Xinpeng Jiang[1], Huan Yuan[1,2], Xin He[1], Te Du[1], Hansi Ma[1], Xin Li[1], Mingyu Luo[1], Zhaojian Zhang[1], Huan Chen[1], Yang Yu[1], Gangyi Zhu[3], Peiguang Yan[4], Jiagui Wu[2], Zhenfu Zhang[1,], and Junbo Yang[1,*]*

[1]*Center of Material Science, College of Sciences, National University of Defense Technology, Changsha 410073, China*

[2]*College of Electronic and Information Engineering, Southwest University, Chongqing, 400715, China*

[3]*Peter Grünberg Research Centre, College of Telecommunications and Information Engineering, Nanjing University of Posts and Telecommunications, Nanjing 210003, China*

[4]*College of Physics and Optoelectronic Engineering, Shenzhen University, Shenzhen 518060, China*

**Corresponding author: yangjunbo@nudt.edu.cn*

zhenfuzhang@nudt.edu.cn

Section S1. Additional information for the theoretical calculation of radiation temperature

As shown in Fig. S1, the experimental device of thermal management includes samples, a glass shield with the thermal barrier film, a heating source, a tunable DC power supply, a thermocouple with a computer monitor. The insulating glass and the thermal barrier film are used for minimizing convection heat transfer and conduction heat transfer. Both of the MFS sample and Au film sample are fabricated on the Si substrate with the same thickness. The dc power supply and the heat source with a resistance of 29 Ω are used for heating the MFS sample and an equal-sized Au film. The thermocouple connects the top surface of sample and tests the heating temperature.

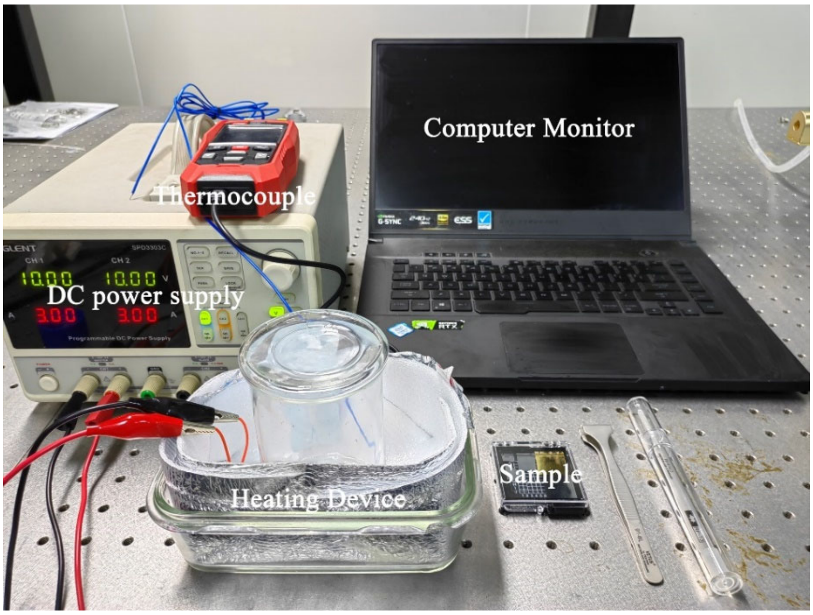

Fig. S1. The experimental device of thermal management includes samples, a glass shield with the thermal barrier film, a heating source, a tunable DC power supply, a thermocouple with a computer monitor.

Section S2. Angle dependence of reflectance spectra for NIR

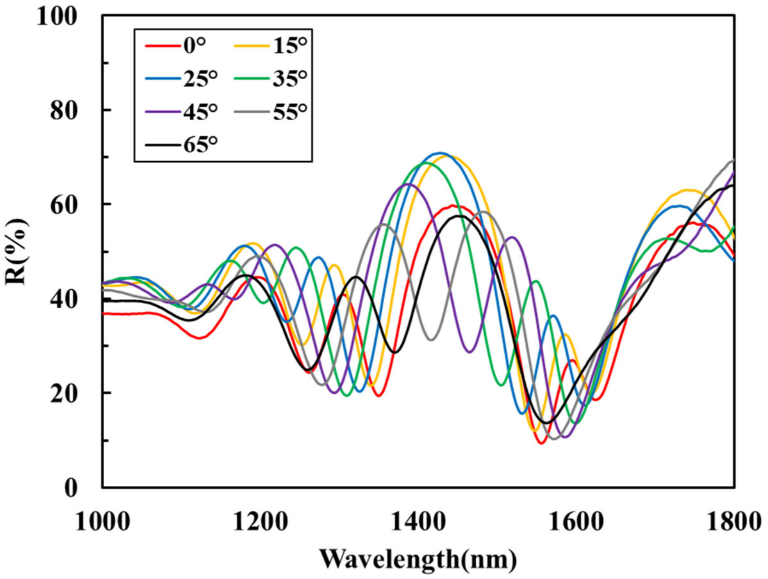

Fig. S2. NIR angle dependence (incident and detection angle: 15° - 65°, with an interval of 10°) of the MFS reflectance spectra from 1μm to 1.8μm.

Section S3. Angle dependence of reflectance spectra for MIR

The angular-dependent MIR spectra were measured by the FTIR (Bruker VERTEX 70v) with a high throughput, variable angle specular reflectance accessory (PIKE Technologies VeeMAX^{TM}) from 40° to 70°. As shown in Fig. S3, the experimental results illustrate the robust incidence independence with the large angle (70°) of the proposed MFS.

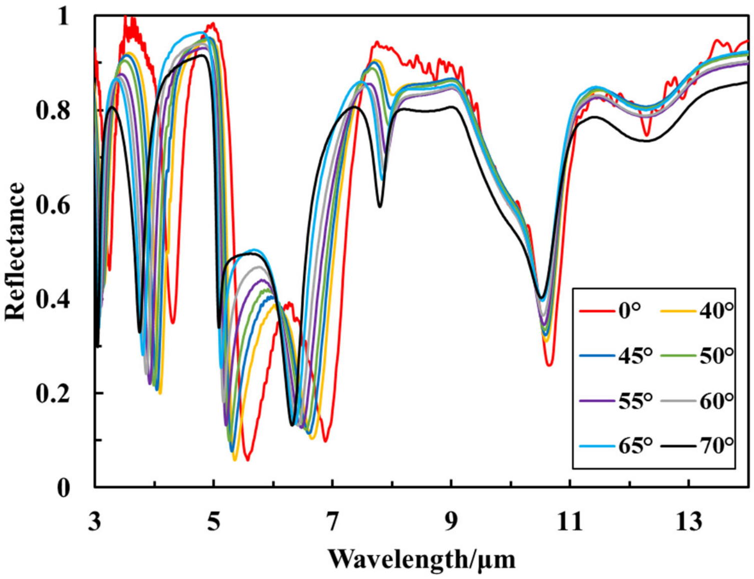

Fig. S3. MIR angle dependence (incident and detection angle: 40° - 70°, with an interval of 5°) of the MFS reflectance spectra from 3μm to 14μm.

Section S4. Comparisons of other metastructures and the MFS for selective emitter

Table S1. Comparison of different dimension of metamaterials for selective absorber(emitter)

Refs.	Materials	Structure	Geometric parameters	Spectral properties	Fabrication
[1]	Polyethylene (PE)/SiO_2	Polymer matrix composites	Different weight ratios of PE and SiO_2 Thickness~840 μm	$\varepsilon_{8\text{–}13\mu m}$>90%	3D printing
[2]	Graphene/SiO_2/Ag	2D gratings	Thickness~90nm	$A_{0.3\text{–}2.5\mu m}$≈85%	Direct laser writing
[3]	Graphene/Cu	3D gratings	Hole width~0.59μm Thickness~30nm Depth~1μm	$A_{0.28\text{–}1.6\mu m}$≈95%	Direct laser writing
[4]	SiN_X/TiN	3D gratings	Thickness~330nm Disk diameter~58nm	$A_{0.25\text{–}2.25\mu m}$≈87%	Photolithography
This work	SiO_2/Ge/ZnS/Pt/Au	Multilayer films (1D)	Thickness~4μm	$A_{1.06\mu m}$≈64% $A_{1.55\mu m}$≈90% $A_{10.6\mu m}$≈76% $A_{5\text{–}8\mu m}$≈54%	Thermal evaporation

References

[1] K. Zhou, W. Li, B. B. Patel, R. Tao, Y. Chang, S. Fan, Y. Diao, and L. Cai, "Three-Dimensional Printable Nanoporous Polymer Matrix Composites for Daytime Radiative Cooling," Nano Lett., 21, 1493−1499 (2021).

[2] K. Lin, H. Lin, T. Yang, and B. Jia, "Structured graphene metamaterial selective absorbers for high efficiency and omnidirectional solar thermal energy conversion," Nat. Commun., 11:1389 (2020).

[3] H. Lin, B. C. P. Sturmberg, K. Lin, Y. Yang, X. Zheng, T. K. Chong, C. M. de Sterke, and B. Jia, "A 90-nm-thick graphene metamaterial for strong and extremely broadband absorption of unpolarized light," Nat. Photonics, 13, 270 (2019).

[4] S. Wu, Y. Ye, Z. Jiang, T. Yang, and L. Chen, "Large-Area, Ultrathin Metasurface Exhibiting Strong Unpolarized Ultrabroadband Absorption," Adv. Optical Mater., 1901162 (2019).
